# Human Endogenous Retroviruses Long Terminal Repeat Methylation, Transcription, and Protein Expression in Human Colon Cancer

**DOI:** 10.3389/fonc.2020.569015

**Published:** 2020-10-27

**Authors:** Maria Dolci, Chiara Favero, Wafa Toumi, Evaldo Favi, Letizia Tarantini, Lucia Signorini, Giuseppe Basile, Valentina Bollati, Sarah D'Alessandro, Pietro Bagnoli, Pasquale Ferrante, Serena Delbue

**Affiliations:** ^1^Department of Biomedical, Surgical and Dental Sciences, University of Milan, Milan, Italy; ^2^EPIGET-Epidemiology, Epigenetics and Toxicology Lab, Department of Clinical Sciences and Community Health, University of Milan, Milan, Italy; ^3^Laboratory Services, Viral and Molecular Tumor Diagnostics Unit, Habib Thameur Hospital, Tunis, Tunisia; ^4^Renal Transplantation, Fondazione Scientific Institute for Research, Hospitalization and Health Care Ca' Granda, Ospedale Maggiore Policlinico, Milan, Italy; ^5^Department of Clinical Sciences and Community Health, University of Milan, Milan, Italy; ^6^Orthopedic Department, San Siro Clinical Institute, Milan, Italy; ^7^Surgical Department, Istituto Clinico Città Studi, Milan, Italy

**Keywords:** human endogenous retroviruses, colon cancer, HERV elements, methylation, HERV expression

## Abstract

Colon cancer is the fourth most common malignancy in both incidence and mortality in developed countries. Infectious agents are among the risk factors for colon cancer. Variations in human endogenous retrovirus (HERV) transcript and protein levels are associated with several types of cancers, but few studies address HERV expression in colon cancer. Fifty-eight patients with advanced-stage colon cancer were enrolled in this study. HERV-H, -K (HML-2), -P LTRs, Alu, and LINE-1 methylation levels and transcription of HERV-H, -K (HML-2), and -P *env* and HERV-K *pol* genes in normal adjacent and tumor tissues were investigated by pyrosequencing and RT-qPCR, respectively. Expression of the HERV-K (HML-2) Pol and Env proteins in selected tissues was examined by Western blotting. Associations between HERV transcript expression and methylation levels and between clinical characteristics and HERV expression were evaluated. Compared to adjacent normal tissues, LINE-1 was hypomethylated in tumor tissues (*p* < 0.05), whereas Alu, HERV-K (HML-2), and -H LTRs showed a decreasing trend in tumor tissue compared to normal tissue, though without a significant difference. The transcription levels of HERV *env* and *pol* genes were similar. However, the HERV-K (HML-2) Pol protein was more highly expressed (*p* < 0.01) in surrounding normal tissues, but the HERV-K (HML-2) Env protein was only expressed in tumor tissues. Although HERV LTR methylation and gene expression did not show significant differences between tumor and normal tissues, HERV protein expression differed greatly. Pol protein expression in normal cells may induce reverse transcription and subsequent integration into the host genome, likely favoring cell transformation; in contrast, the Env protein in tumor tissue may contribute to cancer progression through cell-to-cell fusion.

## Introduction

Human endogenous retroviruses (HERVs) are transposable elements first described more than 30 years ago. It is accepted that HERVs represent relics of ancient exogenous retrovirus infections now integrated in the human genome, fixed in germ cell lines, and vertically transmitted to offspring in a Mendelian manner (1). Currently, the HERV nomenclature is still not standardized (2, 3). However, the traditional criterion used to name HERVs is based on the specific cellular tRNA initiating the reverse transcription reaction. Thus, HERV families are originally named by adding the one-letter code of the amino acid specificity of the most likely tRNA as a suffix to the acronym HERV (4). HERV sequences are composed of four protein-encoding genes (*gag, pro, pol*, and *env*) flanked at the 5′ and 3′ ends by two regulatory regions named long terminal repeats (LTRs) (5). In the course of evolution, those sequences accumulated several mutations that eventually led to gene silencing (1). Under normal conditions, HERV gene expression is highly regulated by multiple mechanisms, such as acetylation and methylation (5). However, abnormal HERV gene and protein expression has been documented in cases of malignancy (6), neuroinflammation (7), and autoimmune diseases (8).

Although controversial, the role of HERVs in cancer development and progression is a major field of research (5). In particular, it is still debated whether HERV dysregulation may be considered a real trigger for carcinogenesis or instead a stochastic effect of the epigenetic alterations commonly observed after cell transformation (9). Regardless, abnormal levels of HERV transcription and protein production have been consistently observed in melanoma (10), seminoma (11), renal cell carcinoma (12), prostate cancer (13), and breast cancer (14). It has also been suggested that some HERV-related proteins may act as cancer-specific antigens and therefore may constitute a novel target for immunotherapy (15). Among all the HERVs, one of the mostly studied for its involvement in the human tumorigenesis is the HERV-K (HML-2) (16). Nonetheless, data with regard to colon cancer are scarce and currently limited to a few reports showing overexpression of HERV-H genes in tumor tissues compared to normal tissues retrieved from negative surgical margins (17). The association between HERV-P and colon cancer was also investigated previously, with opposite findings (18, 19).

The aim of the present study was to assess the putative relationship between HERVs and colon cancer through a comparative analysis of HERV-H, -K (HML-2), and -P *env* and HERV-K (HML-2) *pol* transcription; HERV-K Pol and Env protein expression; and HERV-H, -K (HML-2), and -PLTR methylation levels in malignant tissues and negative surgical margins collected from patients with advanced-stage disease. Additionally, HERV-W was investigated, as control, because of its physiological role and pathological association with diseases different from tumors (20).

## Materials and Methods

### Study Population

Fifty-eight consecutive adult patients with biopsy-proven colon cancer who had undergone surgical treatment at Habib Thameur Hospital in Tunis were enrolled in the study. Specimens (cancer tissues and normal tissues retrieved from negative resection margins) were collected during the operation, immediately stored in RNAlater (Qiagen, Germany) at −80°C, and sent to the Laboratory of Molecular Virology at the University of Milan for analysis. All participants signed an informed consent form. The study was approved by our Institutional Ethics Committee (Comitato Etico Istituto Clinico Città Studi, Ospedale Maggiore Policlinico, Milan, protocol number 683_2017bis) and conducted according to the WMA Declaration of Helsinki.

### HERV-H, -K (HML-2), -P, and -W LTR, Alu, and LINE-1 Methylation Levels

Methylation levels of HERV-H, HERV-K (HML-2), HERV-P, and HERV-W LTRs as well as methylation levels of Alu and LINE-1 sequences were evaluated by pyrosequencing using DNA isolated from the surgical specimens (cancer tissues and normal tissues), as previously described (19, 21, 22).

### HERV-H, -K (HML-2), -P *env*, and HERV-K (HML-2) *pol* Gene Expression

RNA was isolated from 20 mg of cancer tissue and 20 mg of normal tissue retrieved from the negative margins of the same surgical specimen using RNA Blood Mini Kit (Qiagen, Germany) that provides on-column DNase digestion during the RNA extraction process, according to the manufacturer's instructions. One microgram of RNA was reverse transcribed using QuantiTec Reverse Transcription Kit (Qiagen, Germany) according to the manufacturer's instructions.

The sequences of the PCR primers are summarized in Table 1, which also indicates their location and similarity on the human chromosomes (19, 23, 24). The primers were subjected to validation, following the manuscript by Perot and colleagues (25). Briefly, the sequences were aligned using the NCBI Primer-BLAST software (http://www.ncbi.nlm.nih.gov/tools/primer-blast) and checked *in silico* at UCSC (http://genome.ucsc.edu). Then, they were synthetized and purified from Eurofins genomics (Konstanz, Germany). Experimental validations were performed on human genomic DNA by varying the annealing temperature (Tm) from 50 to 60°C, and amplification cycles from 35 to 45 in end-point PCR. The amplicons that showed the right sizes on gel electrophoresis analysis were subjected to Sanger automatic sequencing (Eurofins genomics, Konstanz, Germany).

**Table 1:** Primers set for the real-time PCR amplification.

**Virus**	**Target region**	**Nucleotide**	**Primers**	**Sequences**	**Fragment length**
HERV-H^*^ (23)	*env*	5388–5407	HERV-H Fw	5′-TTC ACT CCA TCC TTG GCT AT-3′	129
		5498–5517	HERV-H Rev	5′-CGT CGA GTA TCT ACG AGC AAT-3′	
HERV-K^§^	*env*	93656–93675	HERV-K Fw	5′-CAC AAC TAA AGA AGC TGA CG-3′	167
		93804–93823	HERV-K Rev	5′-CAT AGG CCC AGT TGG TAT AG-3′	
HERV-K^#^ (24)	*pol*	4212–4232	HERV-K-pol Fw	5′-ATC CCA AAA GAT TGG CCT TTA-3′	166
		4357–4378	HERVK-pol Rev	5′-TTA AGC ATT CCC TGA GGT AAC A-3′	
HERV-P^∧^	*env*	364–382	HERV-P Fw	5′-CAA GAT TGG GTC CCC TCA C-3′	208
		554–572	HERV-P Rev	5′-CCT ATG GGG TCT TTC CCT C-3′	

* *HERV-H env: 100% similarity with Chr.2q24.3*.

§ *HERV-K env: 100% similarity with Chr.12q14.1, and 6q14.1*.

# *HERV-K pol: 100% similarity with Chr. 1q22/23.3; 3q27.2/24/13.2; 5q33.3;5p13.3; 6q14.1; 7q22.2; 7p22.1a/1b; 8q24.3c; 11q22.1; 12q14.1; 19q11; 19p12b/12d/12e; Xq21.33*.

∧ *HERV-P env: 100% similarity with Chr.14q32.12*.

HERV-H, HERV-K (HML-2), HERV-P *env*, and HERV-K (HML-2) *pol* transcription levels were then assessed by relative quantitative real-time PCR using the Rotor-Gene Q instrument (Qiagen, Germany) using the QuantiTect SYBR Green PCR kit (Qiagen, Hilden, Germany), and following the manufacturer's instructions.

The final reaction volume was 20 μl, containing 0.7 μM forward primer (0.2 μM for HERV-K *pol*), 0.7 μM reverse primer (0.2 μM for HERV-K *pol*), and 2 μl of cDNA. The thermal cycle program was as follows: 10 min at 95°C and 45 cycles of 95°C for 15 s, 54°C for 15 s, and 72°C for 20 s. Each sample was tested in duplicate, and no-template controls were included.

Reactions containing the RNA sample but no RT enzyme were added for each sample, to control the potential DNA contribution in the HERV quantification. Ct values for no RT reactions should have been at least five cycles greater than those for the reactions with RT; should the Ct values be <5 cycles greater, the reactions were repeated. The mean expression level of the genes of interest was normalized with that of two housekeeping genes: β-actin and glyceraldehyde 3-phosphate dehydrogenase (GAPDH). Specifically, quantification of HERV *env* and *pol* gene expression was obtained using the relative quantification (RQ) algorithm, as follows:

$$\begin{array}{l} \;\Delta \text{Ct HERV}=\text{ Ct HERV }-\text{ Ct }\left(\text{mean GAPDH and βactin}\right)\\ \Delta \text{Ct mean HERV}=\;\frac{\sum \text{Ct HERV }}{\text{number of samples}}\\ \text{ RQ expression}=\;{2\;}^{-\left(\Delta \text{Ct HERV}-\;\Delta \text{Ct mean HERV}\right)} \end{array}$$

### HERV-K Pol and HERV-K Env Protein Expression

Proteins were extracted from 20 mg of tumor tissue and 20 mg of normal tissue collected from the negative margins of the same surgical specimen. The samples were homogenized using gentleMACS™ Dissociator (Miltenyi Biotec, Bergisch Gladbach, Germany) in lysis RIPA buffer (Thermo Fisher Scientific, United States) with the addition of 3 × Halt^TM^ Protease Inhibitor Cocktail (Thermo Fisher Scientific, United States) and 5 mM EDTA. The homogenized tissues were centrifuged at 4,000 × *g* for 5 min at 4°C to isolate the proteins in the supernatant. Thirty micrograms of protein was separated by SDS-PAGE (BioRad, Italy), and the proteins were blotted onto a nitrocellulose membrane using iBlot 2 Dry Blotting System (Thermo Fisher Scientific, United States) according to the manufacturer's instructions. Overnight incubation with the following primary antibodies was performed: anti-GAPDH (Bio-Techne, United States) diluted 1:7000, anti-ERVK-2 Pol (Novus Biological, United States) diluted 1:1000, and anti-ERVK-7 Env Polyclonal Antibody (Thermo Fisher Scientific, United States) diluted 1:1000. The membrane was incubated for 1 h with secondary antibodies goat anti-mouse IgG peroxidase conjugated at 1:5000 (Thermo Fisher Scientific, United States) and goat anti-rabbit IgG HRP-linked at 1:1000 (Cell Signaling, United States). Both the primary and secondary antibodies were diluted in 5% nonfat dried milk. The chemiluminescent substrate Pierce ECL plus Western blot Substrate (Thermo Fisher Scientific, United States) was added to the membrane, and protein expression was detected following the user protocol.

### Statistical Analysis

Baseline characteristics of the cohort are presented by absolute numbers and frequencies and medians [1st−3rd quartile] for categorical and continuous variables, respectively. The statistical methods used to analyze and compare HERV LTR, Alu, and LINE-1 methylation levels as well as HERV *env* and *pol* gene expression in cancer and normal tissues have been previously reported (19). Possible associations between HERV LTR methylation levels or HERV gene expression in tumor tissues and the baseline characteristics of the patients were also investigated as previously reported (19). HERV-K Pol and HERV-K Env protein expression in cancer and normal tissues was assessed and compared using ImageJ, GraphPad Prism, and paired Student's *t*-tests, as appropriate.

## Results

### Study Population

The baseline demographic and clinical characteristics of the study population are provided in Table 2. Surgical specimens were collected from a cohort of 58 adult patients with colon cancer, with a slight predominance of women (51.7%). The median age at the time of surgery was 63 (Q1–Q3: 50–72) years. The distribution of cancer stage according to the American Joint Committee on Cancer Staging System 7^th^ (26) was as follows: 17/58 (29.3%) stage I, IIA, IIB, or IIC (early stage) and 38/58 (65.5%) stage IIIA, IIIB, IIIC, IVA, or IVB (advanced stage).

**Table 2:** Baseline demographic and clinical characteristics of the cohort of patients enrolled in the study.

	**Total population^**a**^*N* = 58**
**Sex**, ***N*** **(%)**	
Female	30 (51.7)
Male	25 (43.1)
Age at the time of surgery, years median (Q1–Q3)	63 (50.5–72)
**Family history of malignancy**, ***N*** **(%)**	
Yes	8 (13.8)
No	47 (81.0)
**Smoking habit**, ***N*** **(%)**	
Yes	20 (34.5)
No	35 (60.3)
**Alcohol consumption** ***N*** **(%)**	
Yes	6 (10.3)
No	49 (84.5)
**Cancer stage according to AJCC Staging System 7th edition**, ***N*** **(%)**	
Stage IIA, IIB, or IIC	17 (29.3)
Stage IIIA, IIIB, or IIIC	25 (43.1)
Stage IVA or IVB	13 (22.4)
**Cancer location**, ***N*** **(%)**	
Right colon	11 (19.0)
Left colon	44 (75.9)
**Cancer histology according to the WHO classification**, ***N*** **(%)**	
Adenocarcinoma	55 (94.8)
**Chemotherapy**, ***N*** **(%)**	
Yes	20 (34.5)
No	35 (60.3)

a *For three patients (5.2%), there was no information available. Q1, first quartile; Q3, third quartile; AJCC, American Joint Committee on Cancer; WHO, World Health Organization*.

### Alu, LINE-1, HERV-H, HERV-K (HML-2), HERV-P, and HERV-W LTR Methylation

The cancer tissues showed significantly lower mean LINE-1 methylation levels than did the normal tissues, at 73.47% (95% CI: 72.62–74.32) vs. 74.80% (95% CI: 74.11–75.49), respectively (*p* = 0.01). Alu sequences exhibited a similar trend, but the difference between the cancer and normal tissues did not reach statistical significance: 22.34% (95% CI: 22.05–22.63) vs. 22.70% (95% CI: 22.35–23.04), respectively (*p* > 0.05).

**Figure 1:**
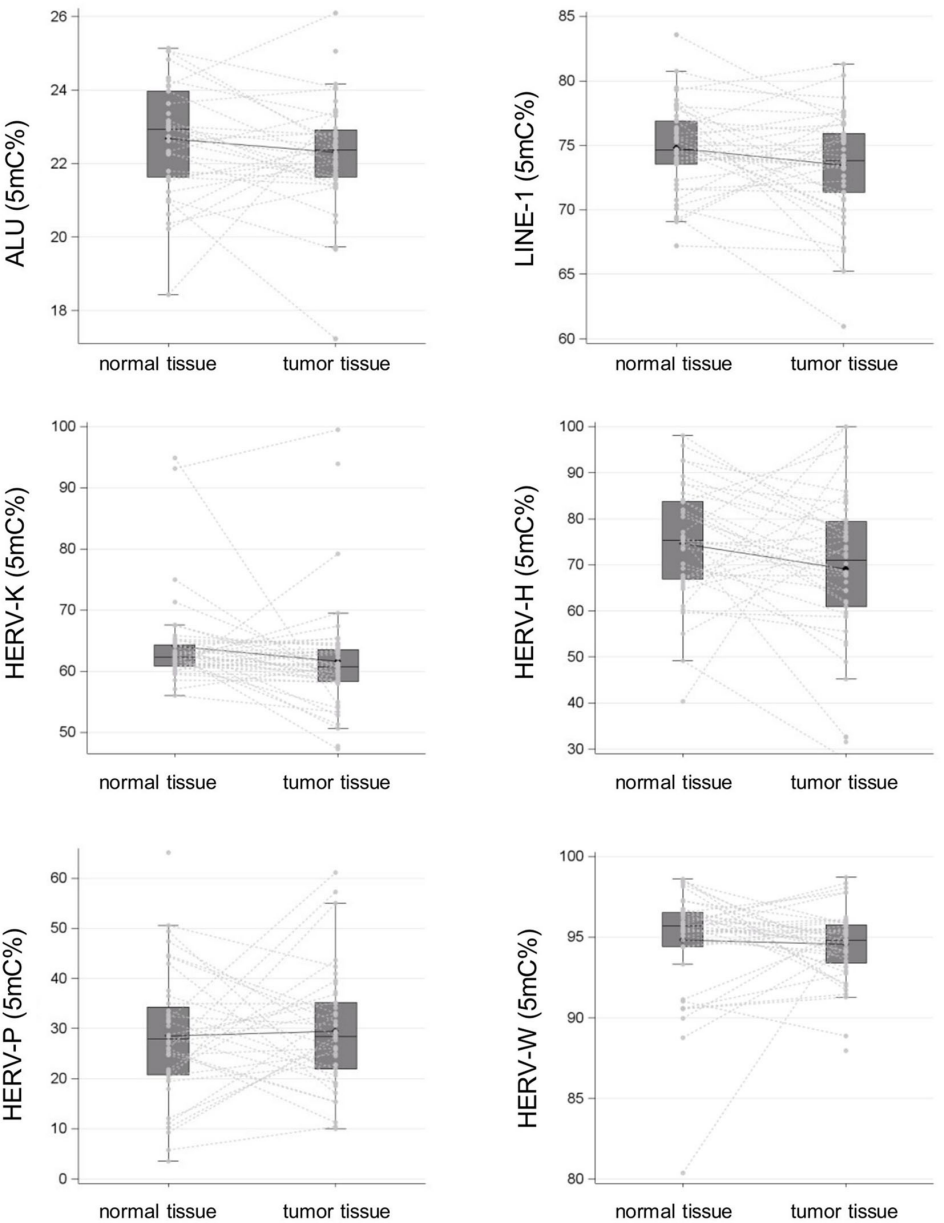
Alu, LINE-1, and HERV-H, HERV-K (HML-2), HERV-P, and HERV-W LTR methylation levels in surgical specimens (tumor tissue vs. normal tissue in negative resection margins) collected from a cohort of patients with colon cancer are reported as spaghetti and box plots.

Additionally, the mean levels of HERV-H and HERV-K (HML-2) LTR methylation were lower in the cancer tissues than in the controls: 69.11% (95% CI: 64.32–73.90) vs. 74.37% (95% CI: 70.41–78.32) and 61.56% (95% CI: 59.15–63.98) vs. 64.20% (95% CI: 62.01–66.39), respectively [*p* > 0.05 for HERV-H and *p* > 0.05 for HERV-K (HML-2)]. In contrast, mean HERV-P and HERV-W LTR methylation levels were comparable in the cancer and normal tissues, at 29.42% [95% CI: 26.06–32.77] vs. 28.43% [95% CI: 24.24–32.62] and 94.56% [95% CI: 93.96–95.15] vs. 94.81% [95% CI: 93.76–95.86], respectively (*p* > 0.05 for HERV-P and *p* > 0.05 for HERV-W). The results of methylation level analysis are illustrated in Figure 1.

### HERV-H, HERV-K (HML-2), HERV-P *env*, and HERV-K *pol* Transcription

**Figure 2:**
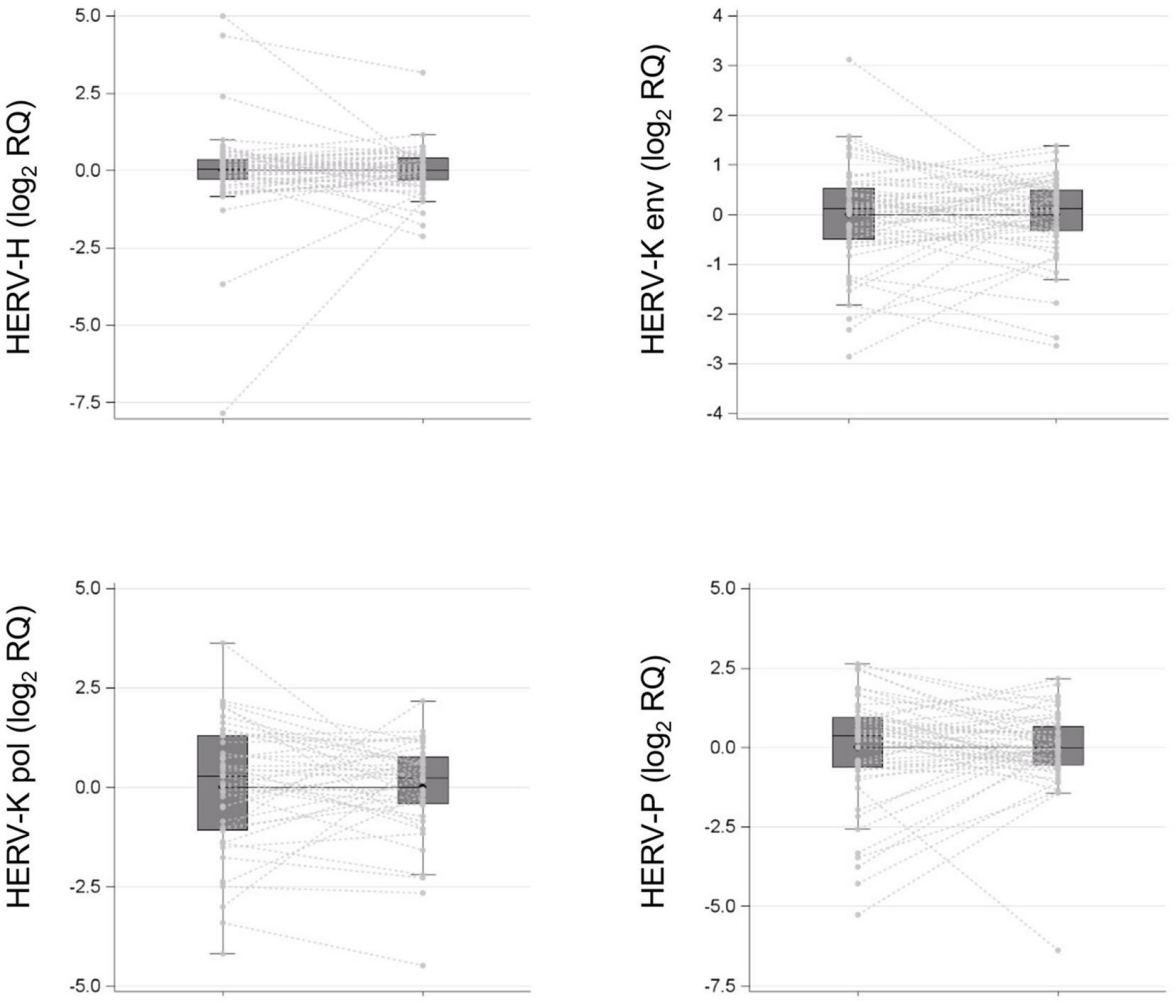
HERV-H, HERV-K (HML-2), HERV-P *env* genes, and HERV-K (HML-2) *pol* gene transcription levels in surgical specimens (tumor tissue vs. normal tissue in negative resection margins) collected from a cohort of patients with colon cancer are reported as spaghetti and box plots.

As depicted in Figure 2, comparison between cancer and normal tissues did not reveal statistically significant differences in HERV-H (1.00, 95% CI: 0.82–1.22 vs. 1.00, 95% CI: 0.66–1.53; *p* > 0.05), HERV-K (HML-2) (1.00, 95% CI: 0.81–1.24 vs. 1.00, 95% CI: 0.76–1.32; *p* > 0.05), or HERV-P (1.00, 95% CI: 0.72–1.40 vs. 1.01, 95% CI: 0.64–1.61; *p* > 0.05) *env* gene or HERV-K (HML-2) *pol* (0.96, 95% CI: 0.66–1.40 vs. 1.03, 95% CI: 0.64–1.65; *p* > 0.05) gene geometric mean transcription levels.

### Correlation Analysis Between HERV LTR Methylation and HERV Gene Transcription

Correlation analysis between the HERV LTR methylation status and HERV transcription levels in surgical specimens is detailed in Table 3. In cancer tissues, we observed a statistically significant association between HERV-P LTR mean methylation levels and HERV-K (HML-2) (correlation value 0.33, *p* < 0.05) or HERV-P (correlation value 0.01, *p* < 0.05) *env* gene geometric mean transcription levels. Furthermore, a statistically significant correlation in normal tissues between HERV-K (HML-2) LTR mean methylation levels and HERV-K (HML-2) *pol* gene geometric mean transcription levels was also noted (correlation value −0.32, *p* = 0.04).

**Table 3:** Spearman correlation between HERV-P, HERV-H, and HERV-K *env* gene geometric mean transcription levels; HERV-K *pol* gene geometric mean transcription levels; and HERV-P, HERV-H, and HERV-K LTR methylation levels in surgical specimens (tumor tissue vs. normal tissue in negative resection margins) collected from a cohort of patients with colon cancer.

	**Normal tissue**^****a****^			**Cancer tissue**		
	**Met HERV-P**	**Met HERV-H**	**Met HERV-K**	**Met HERV-P**	**Met HERV-H**	**Met HERV-K**
rqExpr HERV-P *env*	−0.22	−0.22	−0.11	0.40	−0.07	−0.15
*P-*value	0.15	0.13	0.45	*0.01*	0.68	0.33
Observations (*n*)	45	49	50	38	39	43
rqExpr_HERV-H *env*	0.07	0.26	−0.16	0.05	−0.11	0.16
*P-*value	0.63	0.07	0.27	0.78	0.50	0.31
Observations (*n*)	46	49	52	38	39	43
rqExpr HERV-K_*env*	−0.16	−0.17	−0.23	0.33	−0.03	0.29
*P-*value	0.27	0.24	0.10	*0.04*	0.88	0.06
Observations (*n*)	46	49	52	38	39	43
rqExpr HERV-K_*pol*	−0.11	−0.03	−0.32	0.23	−0.02	−0.03
*P-*value	0.50	0.87	*0.04*	0.19	0.92	0.85
Observations (*n*)	37	39	41	34	35	39

a *Normal tissue collected from negative surgical margins*.

### Association Between Patient Characteristics and HERV *env* and *pol* Gene Expression in Tumor Tissue

No relevant associations between HERV gene [HERV-H, HERV-K (HML-2), HERV-P *env*, or HERV-K (HML-2) *pol*] geometric mean transcription levels in cancer tissues and the baseline characteristics of the study population were observed (Table 4).

**Table 4:** Association between baseline characteristics in HERV-H, HERV-K, and HERV-P *env* genes or HERV-K *pol* gene (measured in tumor tissue). Geometric means were adjusted for age, sex, and smoking status.

		**HERV-H** ***env***				**HERV-K** ***env***				**HERV-P** ***env***				**HERV-K** ***pol***			
		**Mean**	**95% CI**		***P***	**Mean**	**95% CI**		***P***	**Mean**	**95% CI**		***P***	**Mean**	**95% CI**		***P***
Cancer location	Left	0.95	0.73	1.24	0.86	0.92	0.70	1.21	0.44	1.02	0.74	1.40	0.36	0.95	0.56	1.62	0.70
	Transverse	1.13	0.38	3.41		1.46	0.47	4.52		1.40	0.39	4.96		2.99	0.21	42.85	
	Right	1.09	0.66	1.78		1.26	0.76	2.09		1.61	0.91	2.84		0.98	0.36	2.63	
Cancer stage	Advanced	0.92	0.70	1.22	0.41	1,00	0.75	1.34	0.94	1.20	0.86	1.68	0.66	1.18	0.66	2.09	0.38
	Early	1.12	0.77	1.64		1.02	0.68	1.51		1.06	0.67	1.68		0.80	0.40	1.59	
Chemotherapy	+	0.78	0.55	1.12	0.11	0.93	0.63	1.36	0.60	1.16	0.74	1.82	0.96	1.10	0.49	2.44	0.78
	–	1.13	0.86	1.49		1.05	0.78	1.41		1.14	0.81	1.61		0.97	0.57	1.64	
Histologic analysis	Tubular/villous	0.98	0.59	1.64	0.98	0.96	0.56	1.64	0.85	1.02	0.53	1.94	0.68	0.54	0.22	1.32	0.11
	Tubular	0.99	0.77	1.27		1.02	0.78	1.32		1.18	0.87	1.59		1.23	0.74	2.05	

### HERV-K Env and HERV-K (HML-2) Pol Protein Expression

We assessed HERV-K *env* and HERV-K *pol* transcription as well as HERV-K Env and HERV-K Pol protein expression in the surgical specimens (tumor and normal tissues) of seven patients with advanced-stage colon cancer (Stage IIIA, IIIB, IIIC, IVA, or IVB). The characteristics of this subgroup of patients are provided in Table 5. However, as limited samples were available, it was not possible to evaluate protein expression in the other tissues.

**Table 5:** Baseline characteristics of the subgroup of patients with colon cancer selected for HERV-K Env and HERV-K Pol protein expression analysis.

	**Pt 1#**	**Pt 2#**	**Pt 3#**	**Pt 4#**	**Pt 5#**	**Pt 6#**	**Pt 7#**
Sex	Male	Male	Female	Female	Male	Male	Male
Age (years)	68.6	36.9	69.7	89.6	72.1	90	84.1
Cancer location	Left	Right	Right	Right	Left	Right	Right
Histology^a^	Adeno	Adeno	Adeno	Adeno	Adeno	Adeno	Adeno
Cancer stage^b^	III	III	II	IV	III	III	IV

a *Histology according to the colorectal carcinoma World Health Organization classification*.

b *Cancer stage according to the American Joint Committee on Cancer Staging System 7th edition*.

**Figure 3:**
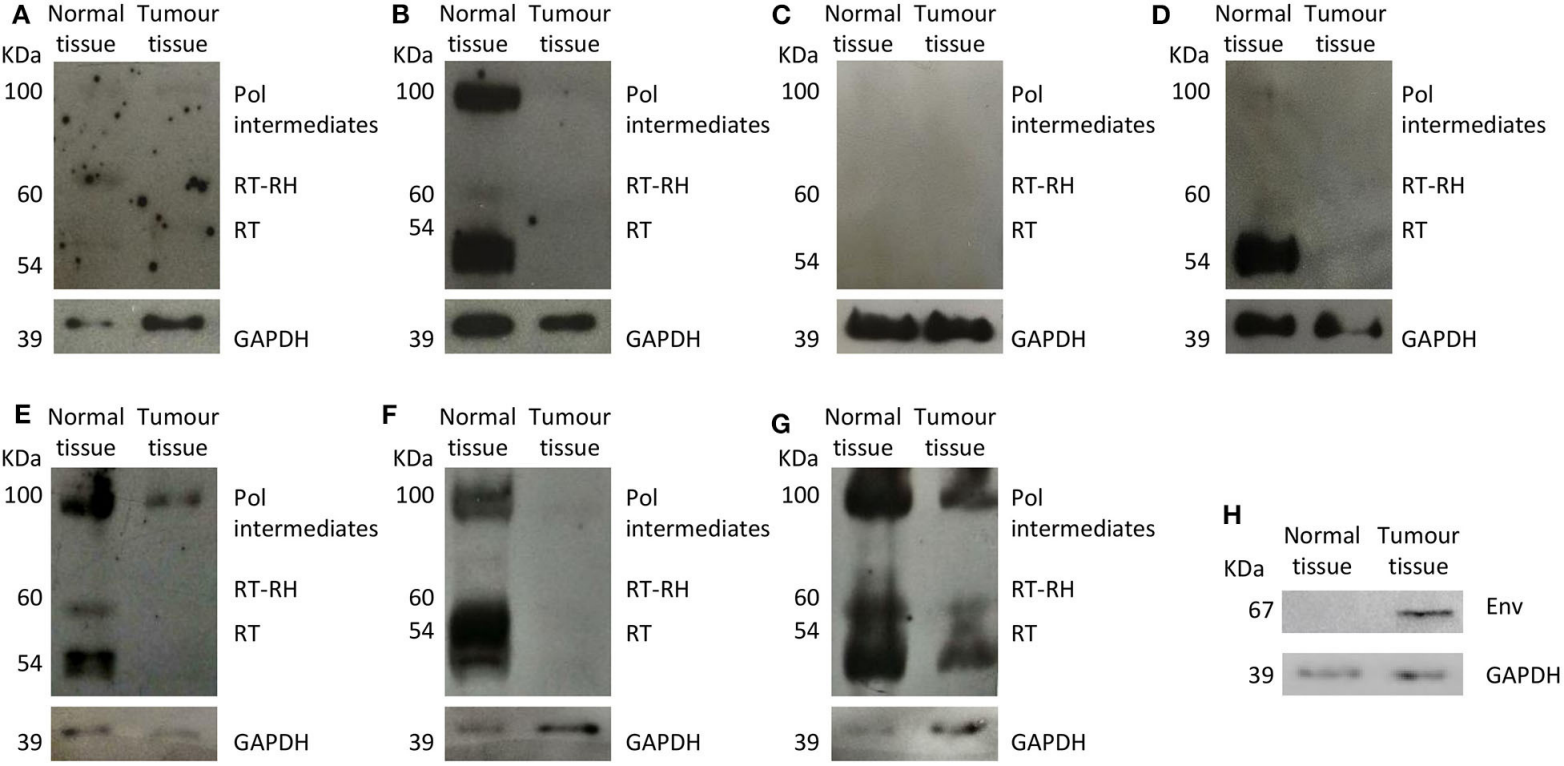
**(A–G)** HERV-K Pol protein expression in colon cancer and normal tissues retrieved from negative surgical margins. HERV-K Pol protein was mainly expressed as three different complexes: polymerase intermediates (Pol, 100 kDa), reverse transcriptase-RNase H (RT-RH, 60 kDa), and reverse transcriptase (RT, 54 kDa). **(H)** HERV-K Env protein (ERVK-7 Env) expression in colon cancer and normal tissues retrieved from negative surgical margins of one patient. GAPDH, glyceraldehyde 3-phosphate dehydrogenase.

Overall, HERV-K *env* and *pol* gene mean transcription levels in cancer tissues and normal tissues were similar (HERV-K *env*: normal tissue 1.19 ± 0.58, and tumor tissue: 1.19 ± 0.71; HERV-K *pol*: normal tissue 2.72 ± 4.42, and tumor tissue 2.23 ± 1.81) (HERV-K env *p* = 0.083 and HERV-K pol *p* = 0.800). Conversely, normal tissues showed significantly higher HERV-K Pol protein expression than did cancer tissues (mean expression levels: 1.97 ± 1.72 and 0.04 ± 0.08, respectively; *p* = 0.001). Intra- and interindividual heterogeneity in the HERV-K Pol protein expression pattern was also noted (Figure 3). In one patient (14.3%), no HERV-K Pol protein expression was detected. Interestingly, HERV-K Env protein expression was restricted to cancer tissues. Moreover, the HERV-K Env protein was not expressed in either normal or cancer tissues in three (42.9%) patients (Supplementary Figure 1).

## Discussion

HERVs are ubiquitous retroviral elements constituting up to 10% of the human genome (27), and these elements have recently been recognized as a potential biomarker in cancer (5, 23, 28, 29). A possible relationship between dysregulation of HERV-related elements and malignancy has also been postulated but not yet confirmed (5).

Several studies, summarized in Supplementary Table 1, reported the possible relationship between HERV expression and tumor, other than colon cancer. In particular, HERV-H was shown to be more expressed in the liver, lung, and testis tumor tissues, compared to normal tissues; similarly, HERV-K (HML-2) gene expression was higher in melanoma, breast cancer, and testis tumor tissues, and HERV-P gene expression was higher in liver tumor tissue.

We instead focused on human colon cancer and assessed the HERV-H, -K (HML-2), and -P LTR methylation status; HERV-H, -K (HML-2), -P *env*, and HERV-K (HML-2) *pol* transcription; and HERV-K Env and Pol protein expression in surgical specimens collected from a population of patients with advanced-stage disease, and the results for the tumor samples were compared to those for normal tissues. Correlation analysis between HERV LTR methylation status and HERV gene expression in cancer tissues was also performed.

These HERVs were chosen due to their possible relationship with cancer: HERV-H, among the known HERVs, is the principal candidate for the colon cancer pathogenesis (17), while the function of the HERV-K HML-2 subtype in carcinogenesis as biomarkers and their potential as targets for cancer are well-described (16). HERV P has been also found differentially expressed in colon cancer tissues (18). Since it is well-established that aberrant DNA methylation contributes to cancer development, and global hypomethylation is generally correlated with tumor grades, in order to verify the specificity of our findings, HERV-W LTR methylation was also tested, as control.

Methylation of LTR regulatory regions is considered one of the main mechanisms responsible for controlling HERV gene transcription in normal cells (5) Indeed, altered LTR methylation has been recently demonstrated in some cancer types, suggesting that it may represent a tumor-specific trait or perhaps a prognostic marker (23, 28, 29). Based on our pyrosequencing analysis, Alu, and LINE-1 methylation levels were significantly lower in tumor tissues than in controls, thus confirming global hypomethylation as a typical characteristic of colon cancer (30). To a lesser degree, we also observed that neoplastic tissues had lower levels of HERV-H and HERV-K (HML-2) LTR methylation than normal tissues but that HERV-P and HERV-W regulatory regions were similarly methylated. In agreement with a previous study (19), these findings suggest that the loss of epigenetic control for HERV-H and HERV-K (HML-2) may be a specific feature of colon cancer and not a consequence of the generalized hypomethylation commonly associated with this type of tumor. The data also indirectly support the hypothesis that dysregulation of HERV-W-related elements may be more appropriately linked to neurological disorders than to malignancy (31).

Current data on HERV gene transcription in colon cancer are quite heterogeneous. As reported by other groups (18, 19, 32, 33), we found that HERV-H, HERV-K (HML-2), or HERV-P *env* and HERV-K (HML-2) *pol* genes were equally transcribed in normal and tumor tissues. A possible explanation is that HERV gene overexpression may be associated or restricted to the early phase of cancer development (25) and that it may eventually disappear when the neoplasm progresses or reaches more advanced stages, as observed in our cohort (19). To this regard, Peròt et al. observed different expression pattern of HERV-H during the different colon cancer phases, with HERV-H overexpression being related to the epithelial to mesenchymal transition (25). In this particular setting, HERV gene upregulation and increased transcription may no longer be necessary because complete malignant transformation has already occurred, following the theory of the “hit and run” tumorigenesis process, postulated for other viruses (34). To validate this theory, future research should include patients with both early- and advanced-stage colon cancer, verifying the different expression of genes and proteins, also comprising gag, with the expectation of high levels of HERV genes and pol proteins in stages 0–2 and low or absent expression in stages 3–4.

Different from the expectation (35), our correlation analysis performed in tumor tissues showed that only HERV-P LTR methylation levels were associated with the corresponding levels of *env* transcription; no relationships for HERV-H and HERV-K (HML-2) were observed. Nevertheless, other studies have reported a lack of correlation between LTR methylation and HERV expression (19). Further correlation analysis between other HERV genes, such as *gag* and *pol*, and LTR methylation is needed to clarify this association.

HERV-K Pol is a protein with different isoforms as a result of proteolytic cleavage (24). However, there is a general lack of knowledge regarding overall and isoform-specific HERV-K Pol expression in human tissues, and its role in tumorigenesis remains mostly unclear (24). According to our analysis, HERV-K Pol expression in colon samples can be highly heterogeneous, with relevant intra- and interindividual variability. Furthermore, our results demonstrate that overall HERV-K Pol expression is significantly higher in normal tissues retrieved from negative surgical margins than in cancer tissues. A disparity in HERV-K Pol variant distribution was also noted because it was mainly expressed in normal tissues as Pol intermediates, complex RT-RH, and RT. These findings are difficult to interpret. A great help in the interpretation could have come from the analysis of the RT activity in the tissues that, due to the paucity of the available materials, was not possible. Thus, we may only hypothesize that HERV-K Pol overexpression in normal tissues surrounding cancer lesions may contribute to genome instability and favor cell transformation through enhanced HERV-K RNA retrotranscription and subsequent integration into the host genome by HERV-K integrase. It can also be postulated that the difference in HERV-K Pol variant expression may be the result of the action of different triggers in normal and tumor cells. Further investigations are needed to clarify the mechanisms regulating HERV Pol expression.

The HERV Env protein consists of two different subunits: a surface subunit that mediates cell adhesion and a transmembrane subunit that possesses immunosuppressive activity and fusogenic properties (36). The importance of HERV Env proteins in physiological settings has been extensively reported. For example, the HERV-W Env protein (namely, Syncytin-1) is primarily involved in the formation of syncytiotrophoblasts (37), and the HERV-FRD Env protein (namely, Syncytin-2) seems to contribute to placental development (38). A potential role of HERV Env proteins in cancer pathogenesis has also been suggested. In their seminal works, Wang-Johanning et al. not only described HERV-K Env protein overexpression in breast cancer (39) but also showed that treatment with specific anti-HERV-K-Env-protein monoclonal antibodies is able to induce apoptosis in malignant cells and to reduce tumor growth (14). Additionally, overexpression of the HERV-K Env protein has been observed in melanoma but not in normal melanocytes or benign melanocyte-derived lesions (40). Data regarding HERV Env expression in colon cancer are currently limited to a single study documenting higher levels of HERV-R Env protein in tumor specimens than in normal tissues surrounding the neoplasms, regardless of tumor grade (32). Similarly, we found that HERV-K Env protein expression is restricted to cancer tissues, even though the HERV-K *env* gene is equally transcribed in normal and neoplastic samples. Considering the specific role of Syncytin-1 and Syncytin-2 in pregnancy and the intrinsic fusogenic potential of the HERV Env protein in general (37, 38), but also taking into account that we did not characterize the expressed proteins, we may only suppose that it may be involved in colon cancer development or progression and represent a potential target for immunotherapy (36).

We recognize that our study has several limitations. First, the population enrolled was relatively small, but a larger cohort of colon cancer patients will be enrolled from patients referring to a hospital from the Milan area, Italy, with particular attention to the colon cancer stages, since most of the patients included in the current analysis had advanced-stage disease. Third, protein expression assessment was limited to an arbitrarily selected pool of surgical specimens.

While the chosen primers for HERV K env and pol were able to anneal to several integrations, the primers for HERV-H and P env had similarity only with the integration on single chromosome. The HERV-H env was selected because it is entirely present on the chr2q24.3. However, it has been reported that HERV-H env fragments can be found on human chromosomes 1, 2, 3, 4, 5, 6, 7, 9, 10, 11, 12, 14, 15, 16, 17, 18, 19, 20, X, and Y, and they all might be subjected to change in the expression. To avoid this limitation, the future project will be focused on more HERV-H insertions, designing a specific PCR for each insertion, so that it could be possible identifying which one might be involved in the colon cancer pathogenesis.

Finally, we did not investigate microsatellite instability, chromosomal instability, or specific gene mutations. Further investigations are warranted to better define both the clinical and translational relevance of HERV-related elements in colon cancer.

## Data Availability Statement

The original contributions presented in the study are publicly available. This data can be found here: NCBI GenBank (https://www.ncbi.nlm.nih.gov/genbank/) accessions: MT992309, MT992310, and MT992311.

## Ethics Statement

The studies involving human participants were reviewed and approved by Fondazione IRCCS Ca' Granda Ospedale Maggiore Policlinico Milano. The patients/participants provided their written informed consent to participate in this study.

## Author Contributions

SD, PF, and WT: conceptualization. MD, LT, LS, and SD'A: methodology and investigation. CF, SD, EF, WT, and PB: data curation. MD, SD, EF, and WT: writing—original draft preparation. EF, GB, PF, PB, and VB: writing—review and editing. PF and SD: supervision and funding acquisition. All authors contributed to the article and approved the submitted version.

## Conflict of Interest

The authors declare that the research was conducted in the absence of any commercial or financial relationships that could be construed as a potential conflict of interest.
