# Supplementary material for: Human Endogenous Retroviruses Long Terminal Repeat Methylation, Transcription, and Protein Expression in Human Colon Cancer
Source: Front Oncol. 2020 Oct 27;10:569015. doi: 10.3389/fonc.2020.569015 (PMC7653092; doi:10.3389/fonc.2020.569015)
Supplement: Supplementary file 1 [file Data_Sheet_1.PDF]

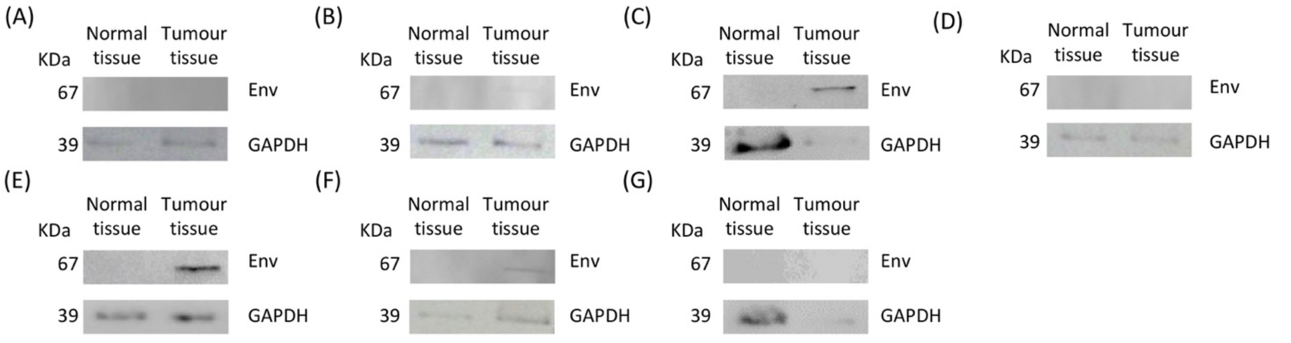


**Supplementary Figure 1**
(A-G) HERV-K Env protein (ERVK-7 Env) expression in colon cancer and normal tissues retrieved from negative surgical margins. GAPDH: glyceraldehyde 3-phosphate dehydrogenase. The HERV-K Env protein was expressed in the samples (B), (C), (E), and (F).

**Supplementary Table 1: Published Manuscripts on the possible association between HERV and cancer**

| Tumors | HERV | RNA and proteins | References |
|---|---|---|---|
| Melanoma | -K<br>-K<br>-K<br>-K<br>-K | Protein (gag, env, rec)<br>Protein (gag, env)<br>RNA, Protein (gag, env, rec) RNA,<br>Protein (env, rec, np9)<br>RNA, Protein (env) | (Muster et al. 2003)<br>(Ishida et al. 2008)<br>(Büscher et al. 2005)<br>(Buscher et al. 2006)<br>(Schiavetti et al. 2002) |
| Breast | -K<br>-K, E, F, W, T, FRD<br>-K<br>-K<br>-K<br>-K | Protein (gag)<br>RNA (pol) RNA (env)<br>RNA, Protein (gag, pol, env)<br>RNA (gag)<br>RNA (env)<br>RNA (env, gag, rec, np9) | (Contreras-Galindo et al. 2008)<br>(Wang-Johanning et al. 2003)<br>(Golan et al. 2008)<br>(Davari Ejthadi et al. 2005)<br>(Johanning et al. 2017)<br>(Tavakolian et al. 2019) |
| Leukemia/ lymphoma | -K<br>-K, -H<br>-K<br>-K<br>-K<br>-E<br>-H | RNA, Protein (gag, env)<br>Protein (gag)<br>RNA (gag)<br>RNA (pol, env)<br>RNA (LTRs)<br>RNA (gag, pol, env)<br>RNA (gag, env) | (Contreras-Galindo et al. 2008)<br>(Sauter et al. 1995)<br>(Ishida et al. 2008)<br>(Iwabuchi et al. 2004)<br>(Simon et al. 1994)<br>(Prusty et al. 2008)<br>(Patzke et al. 2002) |
| Astrocytoma | -K | Protein (env) | (Mameli et al. 2007) |
| Prostate | -K<br>-E, -R | RNA, Protein (gag)<br>RNA (env) | (Ishida et al. 2008)<br>(Wang-Johanning et al. 2003) |
| Lung | -K<br>-E<br>-R<br>-H, -K, -P, -R | Protein (gag)<br>RNA (LTRs)<br>RNA (env)<br>RNA (env) | (Ishida et al. 2008)<br>(Tomita et al. 1990)<br>(Andersson et al. 1998)<br>(Zare et al. 2018) |
| Pancreatic | -K<br>-H | RNA (env)<br>RNA (gag) | (Schmitz-Winnenthal et al. 2007)<br>(Wentzensen et al. 2007) |
| Gastro-intestinal | -K<br>-K<br>-H | Protein (gag)<br>RNA (env)<br>RNA (gag) | (Ishida et al. 2008)<br>(Willer et al. 1997)<br>(Liang et al. 2009) |
| Ovarian | -K<br>-K, -E, -R<br>-E<br>-K<br>-H | RNA, Protein (gag) RNA,<br>Protein (gag) RNA (-)<br>Protein (gag)<br>RNA (LTR) | (Ishida et al. 2008)<br>(Wang-Johanning et al. 2007)<br>(Hu et al. 2006)<br>(Götzinger et al. 1996)<br>(Lower et al. 1993) |
| Endometrial | -W | Protein (env) | (Strick et al. 2007) |
| Testicular, germ cells | -K<br>-K<br>-K, -H<br>-K | Protein (gag, env)<br>RNA (gag)<br>RNA (LTRs)<br>Protein (gag) | (Rakoff-Nahoum et al. 2006)<br>(Herbst et al. 1999)<br>(Vinogradova et al. 2001)<br>(Sauter et al. 1995) |
| Hepatoblastoma | -K | RNA (all genes) | (Grabski et al. 2020) |

## References to supplementary table 1


Buscher, Kristina et al. 2006. "Expression of the Human Endogenous Retrovirus-K Transmembrane Envelope, Rec and Np9 Proteins in Melanomas and Melanoma Cell Lines." *Melanoma research* 16(3): 223–34.

Büscher, Kristina et al. 2005. "Expression of Human Endogenous Retrovirus K in Melanomas and Melanoma Cell Lines." *Cancer Research* 65(10): 4172–80.

Contreras-Galindo, R. et al. 2008. "Human Endogenous Retrovirus K (HML-2) Elements in the Plasma of People with Lymphoma and Breast Cancer." *Journal of Virology* 82(19): 9329–36.

Davari Ejthadi, H. et al. 2005. "A Novel Multiplex RT-PCR System Detects Human Endogenous Retrovirus-K in Breast Cancer." *Archives of Virology* 150(1): 177–84.

Frank, O. et al. 2008. "Variable Transcriptional Activity of Endogenous Retroviruses in Human Breast Cancer." *Journal of Virology* 82(4): 1808–18.

Golan, Maya et al. 2008. "Human Endogenous Retrovirus (HERV-K) Reverse Transcriptase as a Breast Cancer Prognostic Marker." *Neoplasia (New York, N.Y.)* 10(6): 521–33. http://www.ncbi.nlm.nih.gov/pubmed/18516289%0Ahttp://www.pubmedcentral.nih.gov/articlerender.fcgi?artid=PMC2386537.

Götzinger, Nicole, Marlies Sauter, Klaus Roemer, and Nikolaus Mueller-Lantzsch. 1996. "Regulation of Human Endogenous Retrovirus-K Gag Expression in Teratocarcinoma Cell Lines and Human Tumours." *Journal of General Virology* 77(12): 2983–90.

Grabski, David F. et al. 2020. "Upregulation of Human Endogenous Retrovirus-K (HML-2) MRNAs in Hepatoblastoma: Identification of Potential New Immunotherapeutic Targets and Biomarkers: HERV-K Expression in Hepatoblastoma." *Journal of Pediatric Surgery* (xxxx).

Herbst, H et al. 1999. "Human Endogenous Retrovirus (HERV)-K Transcripts in Gonadoblastomas and Gonadoblastoma-Derived Germ Cell Tumours." *Virchows Archiv : an international journal of pathology* 434(1): 11–15.

Hu, Lijuan et al. 2006. "Expression of Human Endogenous Gammaretroviral Sequences in Endometriosis and Ovarian Cancer." *AIDS Research and Human Retroviruses* 22(6): 551–57.

Ishida, Toshiaki et al. 2008. "Identification of the HERV-K Gag Antigen in Prostate Cancer by SEREX Using Autologous Patient Serum and Its Immunogenicity." *Cancer Immunity* 8(November): 1–10.

Iwabuchi, Haruko et al. 2004. "A Gene Homologous to Human Endogenous Retrovirus Overexpressed in Childhood Acute Lymphoblastic Leukemia." *Leukemia & lymphoma* 45(11): 2303–6.

Johanning, Gary L. et al. 2017. "Expression of Human Endogenous Retrovirus-K Is Strongly Associated with the Basal-like Breast Cancer Phenotype." *Scientific Reports* 7(January): 1–11.

Liang, Qiaoyi et al. 2009. "Identification of a Novel Human Endogenous Retrovirus and Promoter Activity of Its 5′ U3." *Biochemical and Biophysical Research Communications* 382(2): 468–72. http://www.sciencedirect.com.pros.lib.unimi.it/science/article/pii/S0006291X09005245?via%3Dihub (September 24, 2017).

Lower, R, J Lower, C Tondera-Koch, and R Kurth. 1993. "A General Method for the Identification of Transcribed Retrovirus Sequences (R-U5 PCR) Reveals the Expression of the Human Endogenous Retrovirus Loci HERV-H and HERV-K in Teratocarcinoma Cells." *Virology* 192(2): 501–11.

Mameli, Giuseppe et al. 2007. "Brains and Peripheral Blood Mononuclear Cells of Multiple

Sclerosis (MS) Patients Hyperexpress MS-Associated Retrovirus/HERV-W Endogenous Retrovirus, but Not Human Herpesvirus 6.” *Journal of General Virology* 88(1): 264–74.

Muster, Thomas et al. 2003. “An Endogenous Retrovirus Derived from Human Melanoma Cells An Endogenous Retrovirus Derived from Human Melanoma Cells.” *Cancer research* 63: 8735–41.

Patzke, Sebastian, Mats Lindeskog, Else Munthe, and Hans Christian Aasheim. 2002. “Characterization of a Novel Human Endogenous Retrovirus, HERV-H/F, Expressed in Human Leukemia Cell Lines.” *Virology* 303(1): 164–73.

Prusty, Bhupesh K et al. 2008. “Transcription of HERV-E and HERV-E-Related Sequences in Malignant and Non-Malignant Human Haematopoietic Cells.” *Virology* 382(1): 37–45.

Rakoff-Nahoum, Seth et al. 2006. “Detection of T Lymphocytes Specific for Human Endogenous Retrovirus K (HERV-K) in Patients with Seminoma.” *AIDS Research and Human Retroviruses* 22(1): 52–56.

Sauter, M et al. 1995. “Human Endogenous Retrovirus K10: Expression of Gag Protein and Detection of Antibodies in Patients with Seminomas.” *Journal of virology* 69(1): 414–21. http://www.ncbi.nlm.nih.gov/pubmed/7983737%0Ahttp://www.pubmedcentral.nih.gov/articlerender.fcgi?artid=PMC188589.

Schiavetti, Francesca et al. 2002. “A Human Endogenous Retroviral Sequence Encoding an Antigen Recognized on Melanoma by Cytolytic T Lymphocytes A Human Endogenous Retroviral Sequence Encoding an Antigen Recognized on Melanoma by Cytolytic T Lymphocytes 1.” *Cancer Research*: 5510–16.

Schmitz-Winnenthal, F. H. et al. 2007. “Potential Target Antigens for Immunotherapy in Human Pancreatic Cancer.” *Cancer Letters* 252(2): 290–98.

Simon, M et al. 1994. “Transcription of HERV-K-Related LTRs in Human Placenta and Leukemic Cells.” *Leukemia* 8 Suppl 1: S12-7.

Strick, Reiner et al. 2007. “Proliferation and Cell-Cell Fusion of Endometrial Carcinoma Are Induced by the Human Endogenous Retroviral Syncytin-1 and Regulated by TGF-Beta.” *Journal of molecular medicine (Berlin, Germany)* 85(1): 23–38.

Tavakolian, Shaian, Hossein Goudarzi, and Ebrahim Faghihloo. 2019. “Evaluating the Expression Level of HERV-K Env, Np9, Rec and Gag in Breast Tissue.” *Infectious Agents and Cancer* 14(1): 1–5.

Tomita, N et al. 1990. “Transcription of Human Endogenous Retroviral Long Terminal Repeat (LTR) Sequence in a Lung Cancer Cell Line.” *Biochemical and biophysical research communications* 166(1): 1–10.

Vinogradova, T. et al. 2001. “Selective Differential Display of RNAs Containing Interspersed Repeats: Analysis of Changes in the Transcription of HERV-K LTRs in Germ Cell Tumors.” *Molecular Genetics and Genomics* 266(5): 796–805.

Wang-Johanning, Feng et al. 2003. “Detecting the Expression of Human Endogenous Retrovirus E Envelope Transcripts in Human Prostate Adenocarcinoma.” *Cancer* 98(1): 187–97.

———. 2007. “Expression of Multiple Human Endogenous Retrovirus Surface Envelope Proteins in Ovarian Cancer.” *International Journal of Cancer* 120(1): 81–90.

Wentzensen, Nicolas et al. 2007. “Expression of an Endogenous Retroviral Sequence from the HERV-H Group in Gastrointestinal Cancers.” *International Journal of Cancer* 121(7): 1417–23.

Willer, A et al. 1997. “Two Groups of Endogenous MMTV Related Retroviral Env Transcripts Expressed in Human Tissues.” *Virus genes* 15(2): 123–33.

Zare, Mokhtar et al. 2018. “Human Endogenous Retrovirus Env Genes: Potential Blood Biomarkers in Lung Cancer.” *Microbial Pathogenesis*.
